# Adenomatous Polyps in Adolescent Girl and Boy: A Report of Two Cases

**DOI:** 10.1155/2016/8256745

**Published:** 2016-10-19

**Authors:** Laleh Vahedi Larijani, Maryam Ghasemi, Hassan Karami

**Affiliations:** ^1^Gastrointestinal Cancer Research Center, Mazandaran University of Medical Sciences, Sari, Iran; ^2^Molecular and Cell Biology Research Center, Mazandaran University of Medical Sciences, Sari, Iran; ^3^Infectious Diseases Research Center with Focus on Nosocomial Infection, Mazandaran University of Medical Sciences, Sari, Iran

## Abstract

A polyp is defined as a mass of the mucosal surface that protrudes into the lumen of the gastrointestinal tract. Neoplastic epithelial polyps are classified histologically as either benign adenoma or malignant carcinoma. The colonic polyps that most commonly present in children occur sporadically and individually and are of the juvenile type; they are most frequently associated with painless rectal hemorrhage (which is the most common symptom). Adenomatous polyps are similar to other nontumoral polyps, and it is very rare for children to have symptoms other than rectal bleeding. This report describes two rare cases of polyps in pediatric patients. An 11-year-old girl presented with tubulovillous adenoma and a 13-year-old boy with tubular adenoma; both patients complained of rectal hemorrhage as well as anemia and abdominal pain. Epithelial adenoma is a tumor that is rarely found in adults or children. Colonoscopic perforation and biopsy are mandatory for establishing a definitive diagnosis and avoiding medical mismanagement.

## 1. Background

Polyp is a tumoral mass that protrudes into the lumen of the digestive tract. Histologically, colorectal polyps may be classified as epithelial or nonepithelial and neoplastic and nonneoplastic type [1].

Most epithelial polyps in children are classified as nonneoplastic, single, and sporadic. Juvenile polyps are the ones more commonly found in children and are reported to account for 84% to 97% of pediatric cases of polyps. They occur most commonly in children 2 to 6 years of age and are slightly more frequent in males than in females [1–6].

Other types of nonneoplastic epithelial tumors include hyperplastic and inflammatory polyps. Some studies report that the combined prevalence in both men and women is 10% [6], and some textbooks report the prevalence of hyperplastic polyps to be 3% [7].

There is little tendency for recurrence in any type of nonneoplastic epithelial tumors, and their progression to malignancy is virtually nonexistent [8, 9]. Their size range is from about 3 to 5 mm [1]. A single adenomatous polyp is a neoplastic polyp that occurs very rarely in children. Pathologically, it is classified as tubular adenoma, tubulovillous adenoma, and villous adenoma [1, 5, 10].

The presence of dysplasia in cells of this type of tumor differentiates it from the nonneoplastic types. The dysplastic changes include nuclear hyperchromasia, abundant mitosis, and loss of polarity, and the tumors are categorized from low- to high-grade based on the severity of these changes. If the tubule formation is more than 75%, the tumor is categorized as tubular adenoma, and if the villous component is more than 75%, the tumor is called villous adenoma. If the content of villous and tubule is 50% it is called tubulovillous adenoma [1].

Adenomas are capable of progressing to malignancy, the process of which requires 7 to 10 years on average. Rectal polyps may occur in children who have polyposis syndromes, including the adenomatous or hamartomatosis syndromes [1].

All these familial polyposis syndromes may predispose the patient to malignancy [4, 8, 9]. Clinically, the diagnoses of the syndromes are based on the clinical picture and the colonoscopy and endoscopy findings [11, 12]. Therefore, rectal hemorrhage in children must always be taken seriously, and the possibility of polyposis syndromes should only be ruled out after careful study. Otherwise, the patient could have prophylactic therapies, such as colonoscopy [13].

Polyps of the colon, whether single or multiple, sporadic or familial, or neoplastic or nonneoplastic, will manifest with abdominal cramping and pain, mucous diarrheal stool, rectal prolapse, and (in long-standing cases) anemia [1, 4].

These symptoms, however, may be found in other disorders such as bacterial or amoebic dysentery, ulcerative colitis, and other inflammatory conditions. Colonoscopy with biopsy will distinguish between these disorders [13].

## 2. Case Presentation

The first patient was a 13-year-old boy who presented with symptoms of mucous diarrheal stool, cramps, and fresh blood discharge as well as hypochromic microcytic anemia that had started 4 to 5 months earlier (Table 1).

**Figure 1:**
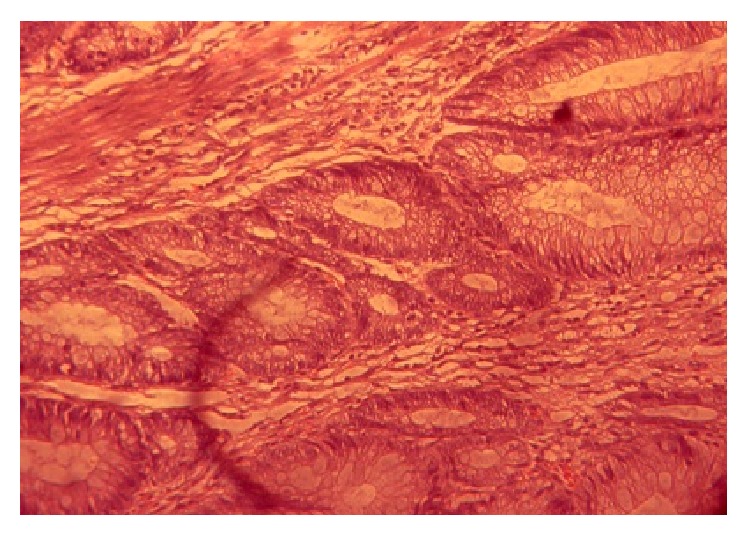
Tubular adenoma (case 1)—high power.

**Figure 2:**
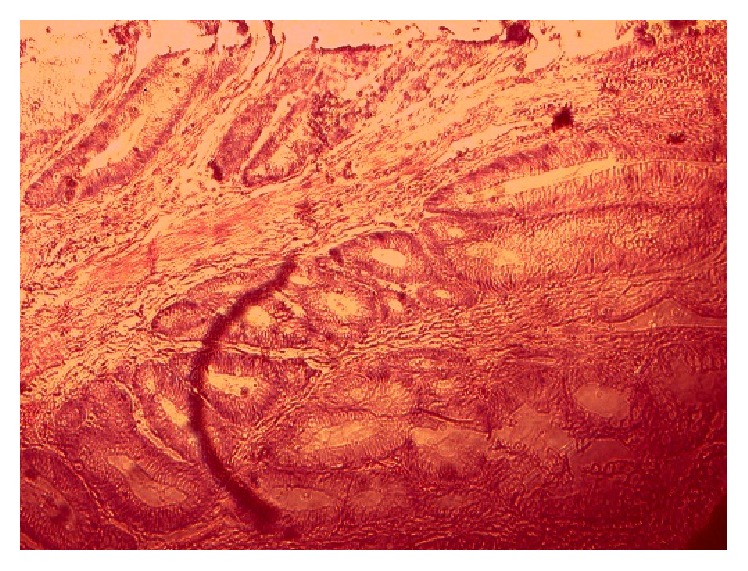
Tubular adenoma (case 1)—low power.

The patient had no history of polyposis syndromes or adenocarcinoma of the digestive tract or of any family history of breast tumor, ovarian tumor, or brain tumor. The colonoscopy revealed a sessile, cauliflower-shape, deformed polypoid mass in the rectum with the appearance of an adenocarcinoma. The patient underwent surgery, and the tumor was removed. The surgery reported a polyp measuring 5 × 4 × 3 cm located at 3 cm along the pectinate line. Specimens were obtained from II, III, and VI o'clock positions. Microscopy showed the hyperplastic structures of the tubular glands standing back-to-back on an inflammatory and vasculated background. The cells contained enlarged, elongated nuclei and lost polarity, with increased chromatic density, prominent nucleoli, and abundant mitosis. Some parts of the polyp were ulcerated. However, there was no sign of invasion to the submucosal tissue, and a diagnosis of tubular adenoma was established (Figures 1 and 2).

The second patient was an 11-year-old girl with hematochezia (passage of fresh blood from the rectum) that had begun 6 months earlier. The clinical history included no mention of any polyposis syndrome in the patient's family.  Table 2 shows the results of the test.

**Figure 3:**
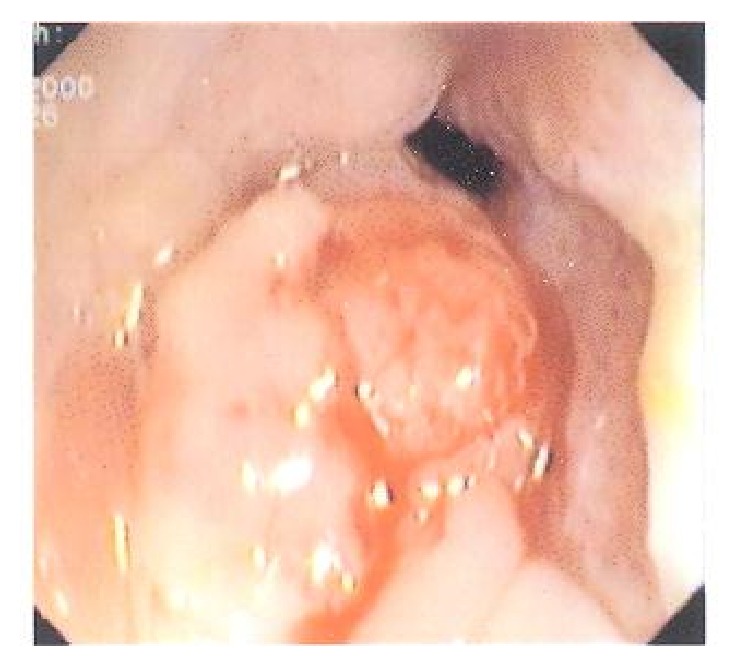
Tumoral mass in rectum (case 2).

**Figure 4:**
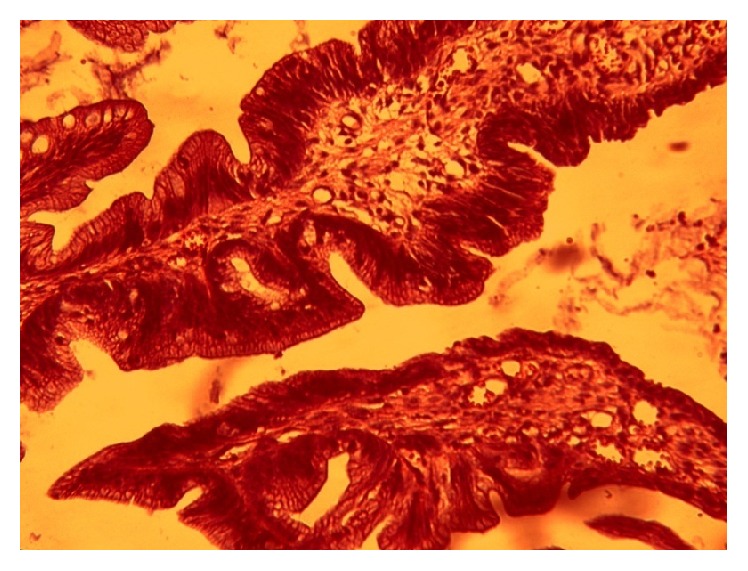
Villous adenoma (case 2).

In order to identify the cause of the hemorrhage, we performed a barium transit study, an abdominal and pelvic CT scan, and sonography and took the measurements for lactate dehydrogenase (LDH), sedimentation, aspartate aminotransferase (AST), alanine aminotransferase (ALT), and alpha fetoprotein (*α*FP), which were normal. Colonoscopy revealed ulcerating and scaring in certain areas of the rectum as well as a sessile (Figure 3). The patient was sent to surgery for sampling or excision with a suspicion of adenocarcinoma. Surgery reported a cauliflower-shape, polypoid, sessile mass measuring 4 × 4 × 3.5 cm located at 4 cm along the pectinate line. Microscopy revealed that the tumor consisted of back-to-back villous and tubular structures with the former constituting more than 75% of the mass. The covering cells had lost polarity and displayed long, vesicular nuclei, prominent nucleoli, and abundant mitosis. No stromal invasion was found and, therefore, a diagnosis of villous adenoma was established (Figure 4).

## 3. Discussion

The most common symptoms of polyps in children are fresh bleeding from the rectum with no pain [1–4]. The other disorders of the digestive tract such as ulcerative colitis and bacterial and amoebic dysentery may also cause these symptoms. Other less common symptoms include abdominal pain, nausea, vomiting, mucus passage, and anemia [1, 4].

A number of different studies have emphasized the importance of investigating fresh bleeding that occurs from the rectum in children and the need for colonoscopy. It has been observed that many pediatric polyps have been untreated because of insufficient attention to rectal hemorrhage, leading to irreversible complications for the patient. While the clinical symptoms of neoplastic and nonneoplastic polyps, as well as those of the syndromic types, are similar the treatment and follow-up are quite different and play a crucial role in the patient's prognosis [3].

Based on the importance of these factors, we have reported two very rare cases of polyp: tubular adenoma and tubulovillous adenoma in adolescent boy and girl. Both patients, have had fresh blood passage from the rectum for 6 months with abdominal pain and anemia. Neither had a history of any polyposis syndrome in their families and no signs of syndromic disorders were found on physical examination. Colonoscopy revealed large (4 to 5 cm) cauliflower-shaped masses at 4 cm along the pectinate line, raising the suspicion for adenocarcinoma. Pathologic finding diagnosed tubular and tubulovillous adenoma. The aim of this report was to emphasize the importance of hematochezia in children and the need for colonoscopy. A complete and thorough examination of the patient will help to prevent errors in diagnosis that could lead to catastrophic results for the patient.

## Figures and Tables

**Table 1:** Results of first test in case 1.

S/E	WBC: many RBC: 15–20	

S/C	Negative	

CBC	WBC: 4 × 10^3^ Hb: 9.3 Hct: 28.3	MCV: 70 MCH: 23.3 MCHC: 31 Plt: 234 × 10^3^

**Table 2:** Results test in case 2.

S/E	WBC: 0–10 RBC: 15–20	

CBC	WBC: 8.1 × 10^3^ RBC: 2.7 × 10^6^ Hb: 9.5 Hct: 28.5	MCV: 72 MCH: 24 MCHC: 31.84 Plt: 596 × 10^3^
